# Legume Consumption Patterns in US Adults: National Health and Nutrition Examination Survey (NHANES) 2011–2014 and Beans, Lentils, Peas (BLP) 2017 Survey

**DOI:** 10.3390/nu12051237

**Published:** 2020-04-27

**Authors:** Thushanthi Perera, Candace Russo, Yumie Takata, Gerd Bobe

**Affiliations:** 1 College of Public Health and Human Sciences, School of Biological and Population Health Sciences, Oregon State University, Corvallis, OR 97331, USA; pererah@oregonstate.edu (T.P.); yumie.takata@oregonstate.edu (Y.T.); 2 Linus Pauling Institute, Oregon State University, Corvallis, OR 97331, USA; candace.russo@oregonstate.edu

**Keywords:** legumes, cross-sectional survey, NHANES

## Abstract

Given the emerging health benefits of regular legume consumption, we hypothesized that the historically low legume consumption levels in US adults increased. We evaluated legume consumption patterns in US adults using cross-sectional data from the 2011–2012 and 2013–2014-year cycles of National Health and Nutrition Examination Surveys (NHANES) and a 2017 cross-sectional, online survey of Oregon families named “Beans, Lentils, Peas (BLP) Survey”. We also compared legume consumption patterns between consumers below US dietary recommendations for mature legumes (<37.5 g/day, marginal), below levels showing nutritional and disease-prevention benefits (37.5–87.49 g/day, recommended); and levels demonstrating nutritional and disease prevention benefits (≥87.5 g/day; beneficial). In NHANES, legume consumption remained low in US adults and declined from 2011 to 2014 (mature legumes: 12.8 to 8.3%; dry beans: 10.0 to 6.5%). In BLP, less than 5% consumed legumes daily; approximately one-third did not consume legumes during the last month. Marginal mature-legume consumers ate a limited variety of legumes (dry beans and green legumes on a weekly to monthly basis). Beneficial amount consumers ate mature legumes daily or every other day and included chickpeas, lentils and dry peas to their legume mix. Our data suggest that legume consumption declined in US adults, warranting improved communication about the benefits of regular legume consumption.

## 1. Introduction

Legumes are defined as the immature, germinated, or mature edible seeds of legume plants (Fabaceae) [1]. The primarily consumed legumes are dry beans, dry peas, lentils, chickpeas, cowpeas, fava beans, and pigeon peas, which are grouped as mature legumes, green beans and peas, which are considered immature legumes, and sprouted or germinated beans and peas, which are considered sprouted legumes [2]. Soybeans and peanuts are usually excluded, because of their higher lipid content [1]. Legumes are an important part of a daily diet because of their nutritional and health benefits [3]. They are a good dietary source of protein, fiber and folate, are very low in saturated fatty acids, cholesterol, and sodium, and contain many non-nutritive bioactive components [4]. Consuming 0.5 cup/day (87.5 g) of mature legumes increased fiber, protein, folate, zinc, iron, and magnesium intake and decreased saturated and total fat intake in US adults according to the nation-wide, cross-sectional National Health and Nutrition Examination Surveys (NHANES) 1999–2002 [5]. Based on their nutritional benefits, the current Dietary Guidelines for US Americans consider mature legumes as a food group with a weekly recommendation of 1.5 cups (equivalent to 37.5 g cooked mature legumes/day) for non-vegetarians and 3 cups (equivalent to 75 g cooked mature legumes/day) for vegetarians [6]. In addition to their nutritional benefits, daily mature legume consumption may have disease prevention benefits, specifically, preventing or managing obesity, metabolic syndrome, cardiovascular diseases, and colorectal cancer, as concluded by several meta-analyses [4,7,8,9,10]. In NHANES 1999–2002, dry bean consumption was associated with low body weight, decreased systolic blood pressure, and a smaller waist circumference in US adults [11].

The most recently published legume consumption data for US adults are from the NHANES 1999–2002 two-year cycles, in which 7.9% of US adults consumed mature legumes [5] and 11.1% consumed dry beans on a given day [11]. An earlier study using the Continuing Survey for Individual Intakes (CSFII) 1994–1996 data reported that 14% of the US adults consumed dry beans on two given days [12]. Given the health benefits of regular legume consumption published in the last 15 years, we hypothesized that legume consumption increased in US adults, counteracting the previous trend of a consistent decline in legume consumption. Thus, the objective of this report was to evaluate current legume consumption patterns in US adults. To accomplish our objective, we used data from the 2011–12 and 2013–14-year cycles of NHANES, as more recent NHANES data have not been released yet. To have an estimate of more recent legume consumption, we conducted, in fall 2017, a cross-sectional, online survey of families around Corvallis, Oregon [“Beans, Lentils, Peas (BLP) Nutrition Education Online Parent Survey”]. The BLP survey complemented the findings of the NHANES survey, as the BLP survey asked for consumption of legume groups and their primary dish types during the last month, whereas NHANES provided legume consumption data from 24 h food records for two non-consecutive days as food codes, some of which did not specify legume group and dish type.

## 2. Materials and Methods 

### 2.1. Design and Data Collection

The NHANES is a cross-sectional, representative, nation-wide survey with the objective to monitor the health and nutritional behavior and status of the US civilian, non-institutionalized population [13]. The NHANES has been conducted every year since 1959. Data has been released for two years combined, with the last data being released for the 2013–2014-year cycle. Dietary information is collected by an interviewer-administered 24 h dietary recall in a mobile examination center and a telephone 24-h dietary recall conducted three to ten days after the interview using an automated multiple-pass method [14]. A status code is assigned to each dietary interview to indicate the quality and completeness of the data; only data with complete and reliable dietary records were included in this analysis. Detailed descriptions of the dietary interview methods are provided in the NHANES Dietary Interviewers Procedure Manuals [13]. Besides nutrition, health, and demographic information, NHANES collects information from standardized physical examinations and laboratory tests by trained staff [14].

The BLP was a cross-sectional online survey that contained two parts: (1) quantify recent legume consumption patterns and (2) identify the rationale (i.e., benefits and barriers) for their legume consumption pattern. In this report, we primarily focused on the first part. An e-mail with the electronic link to the online survey was sent over the healthy youth program (HYP) listserv, which reaches HYP participants who are children and their parents or guardians. The HYP is an outreach branch of the Linus Pauling Institute of Oregon State University, which provides evidenced-based cooking, nutrition, and garden-based hands-on education program to families and communities around Corvallis, Oregon (http://lpi.oregonstate.edu/healthyyouth). The HYP listserv included a representative sample of families in Oregon with various socio-economic backgrounds. The survey was initiated on November 15, 2017, and closed on December 13, 2017. Two e-mail reminders were sent on November 27 and December 4 to encourage participation. The survey was sent to 949 e-mail addresses of the HYP listserv. The electronic link to the survey was uploaded by 250 e-mails from 194 families. Our statistical analysis included the surveys that were over 40% completed (143 out of 164 started surveys). The study was reviewed and approved by the Institutional Review Board of Oregon State University (IRB protocol number 8303 “Beans, Lentils, Peas (BLP) Nutrition Education Online Parent Survey”).

### 2.2. Instrument

Data from the NHANES 2011–2012 and 2013–2014-year cycles were used in this study. The combined 4 years were limited to adults aged >18 years, excluding women who were pregnant or lactating at the time of data collection. We used the United States Department of Agriculture (USDA) Food and Nutrient Database for Dietary Studies, 5.0 (FNDDS) to identify all food sources of legumes, including mixed dishes and recipes [15]. Legumes were subdivided into nine groups: dry beans (e.g., pinto, pink. navy, kidney, mung, lima, and black beans), dry peas (e.g., yellow and green split peas), lentils, green legumes (e.g., green beans and green peas), sprouted legumes (sprouted beans and peas), chickpeas, cowpeas, pigeon peas, and fava beans (including mixed dishes and recipes). Soybeans and peanuts were excluded because of their higher lipid content compared with the other legumes. For the analysis, we selected, from a total of 8600 food codes, the 322 of the 513 legume-related food codes that had at least one positive response (219 of 289 in the 2011–2012 cycle and 305 of 481 in the 2013–2014 cycle). For calculating legume intake, we accounted for the legume proportion in mixed dishes and recipes and used the weight of cooked legumes. For calculating legume dish type frequency, we assigned: (1) side dish to food codes that had legumes as sole descriptor or combined with another vegetable; (2) main dish to food codes that include legumes as part of a main dish (combined with rice/meat/stew/chili); (3) soups to food codes that include legumes as part of a soup; (4) salads to food codes that include legumes as part of a salad; (5) dips to food codes that include hummus or legumes as part of a dip or sauce; and (6) breakfast items, snacks, baked goods, and desserts to food codes that include legumes as part of breakfast items, snacks, baked goods, and desserts.

An online survey was developed with the assistance of professionals of the HYP and Oregon State University nutrition education staff, who had previously worked with the survey participants for program evaluations. The survey was voluntary, online, in English only, and anonymous, and contained 12 questions. The length of our online survey was kept to a minimum (the goal was to keep the survey <10 min, which was achieved by 71% of respondents; the median time of completion was 8 min and 46 s). Two questions queried demographic information (age range and sex). To maintain confidentiality, we did not ask about race, ethnicity, social economic status, and specific age. One question asked recent legume consumption (“How often and how much of the following legume types did you eat in the last month?”), including legume group (green beans/peas, sprouted beans/peas, dry beans, dry peas, chick peas/garbanzo beans, lentils, cow peas/black-eyed peas, fava/broad/horse beans), average serving size of cooked legumes [none, ¼ cup (43.75 g), ½ cup (87.5 g) and 1 cup (175g)], consumption frequency (≥2 times per day, 1 time per day, 5–6 times per week, 3–4 times per week, 2 times per week, 1 time per week, 2–3 times per month, 1 time per month, less than 1 time per month; none), and “most common dish with legumes” (soups, salads, dips, main dish, stand-alone side dish, breakfast item, desserts, and baked goods, others, none). The serving size and consumption frequency categories were adapted from the Polyp Prevention Trial questionnaire [16,17]. The most common dish types were adapted from categories by FAO [18] and Pulse Canada [19]. The remaining questions were formulated to assess the second objective [20]. The survey was previewed and completed by a subset of families. In addition, we queried respondents regarding challenges and preferences associated with the survey. The survey was not pre-tested for its validity. Legume consumption estimates in both surveys were based on cooked legumes, as the serving size of cooked immature legumes is roughly equal to the serving size of raw green or sprouted legumes.

### 2.3. Statistical Analysis

All statistical analyses were performed using STATA 12 and SAS version 9.4 software (SAS Institute, 2013). To identify characteristics of any legume consumers, we compared any legume consumers and non-consumers, defined as those respondents who reported consuming or not consuming any legumes (mature, green, and sprouted) during the 2-day food records for the NHANES data and during the last month for the BLP, respectively. To compare legume consumers by consumption level, we switched from any to mature legume consumption level, as dietary recommendations only exist for the latter. To identify characteristics that differentiate between mature-legume consumers that consume low amounts, those that consume amounts sufficient to fulfill dietary recommendations (37.5 g/day is the current dietary recommendations for cooked mature legume intake for non-vegetarians [6]), and those that consumed amounts needed to alter nutrient profile and show disease prevention benefits (87.5 g/day is amount needed to alter nutrient profile [5] and show disease prevention benefits [4,7,8,9,10]), we compared marginal consumers (0.1–37.5 g cooked mature legumes/day), recommended consumers (37.5–87.49 g/day), and beneficial amount consumers (≥87.5 g/day), respectively. The NHANES analyses used weights designed to account for the unequal selection probabilities, adjustments to independent population controls, and nonresponse. Post-stratification weights were used to adjust sample estimates based on design features of the study and nonresponse. Legume consumer groups were compared using a *t*-test (for comparison of any legume consumers vs. non-consumers) or generalized least-squared means (for comparison among mature legume consumer groups) for continuous data and a chi-square test (NHANES) or Fisher’s exact test (BLP) for categorical data. All tests were two-sided. Significance of group differences was determined to be at *p* ≤ 0.05.

## 3. Results

To identify the characteristics of legume consumers, we compared any legume consumers and non-consumers (Table 1). In the NHANES surveys, age-associated legume consumption had a bell-shaped curve with the highest proportion of legume consumers between the age of 46 and 55 years and the lowest between the age of 18 and 25 years. A similar bell-shaped curve was observed in the 2017 BLP survey. There were no overall gender differences between legume consumers and non-consumers in the NHANES and BLP surveys; however, the age-associated bell-shaped curve was more distinct for men versus women in the NHANES surveys. People of Hispanic descent (Mexican and other Hispanic) had the highest proportion of legume consumers in the NHANES surveys, whereas a smaller proportion of non-Hispanic black and white people consumed legumes. Education level had a significant effect on legume consumption, with consumers in the above and below high school categories being overrepresented. Caloric intake was higher in legume consumers than in non-consumers. Household income did not significantly affect legume consumption. Characteristics were similar for total and mature legume consumption (results not shown).

In the NHANES, legume consumption frequency in US adults was low and showed a downward trend from 2011 to 2014 of 18.5% to 13.7% on a given day (Table 2). The downward trend was primarily due to a decrease in dry bean consumption from 10.0% to 6.5%. The primary legume groups consumed were dry beans and green legumes; chickpeas and lentils were consumed by about 1% of participants on any given day. Very few NHANES participants consumed cowpeas (0.27%), dry peas (0.20%), sprouted legumes (0.13%), pigeon peas (0.02%), or fava beans (0.01%) on a given day. The primary dry bean types consumed were reported as pinto beans (23.1% of total dry bean frequency), followed by refried beans (14.0%; usually pinto beans), black beans (13.7%), red kidney beans (8.3%), baked beans (7.2%; usually navy beans), red beans (4.5%), and white beans (2.4%); the remaining dry bean types contributed each to less than 2.0% of the total dry frequency. The dry bean type was not specified for 21.3% of the total dry bean frequency, which were primarily soups and bean dishes combined with rice or meat.

The results from the BLP survey were similar to those of the NHANES (Table 2). In NHANES, 5.6% (2011–2012-year cycle) and 4.0% (2013-2014-year cycle) of participants ate legumes on each day of data collection compared with 4.9% of participants in the 2017 BLP survey. Similar to NHANES, the most consumed legume types in the BLP 2017 survey were dry beans followed by green legumes, chickpeas, and lentils. Sprouted legumes (21.7%), dry peas (18.9%), and cowpeas (14.0%) were eaten during the last month by less participants.

Legumes were mainly consumed as side or main dish (both primarily dry beans and green legumes) by NHANES participants (Table 3). Other dish types of legume intake were soups (primarily dry beans and lentils), dips (primarily chickpeas as hummus and dry bean dips), and salads (primarily sprouts and green legumes). Breakfast items (0.13%), baked goods (0.13%), snacks (0.03%), and desserts (0 respondents) were rarely a source of legume consumption. A declining trend over time was observed for using legumes as soup and main dish. Results in the BLP survey were similar to those in NHANES (Table 3), as the primarily reported dishes containing legumes were main and side dishes and, to a lesser extent, soups, dips, and salads. Breakfast items (1 respondent), baked goods (1 respondent), and desserts (0 respondents) were rarely a source of legume consumption.

Lentils and dry beans were consumed at higher quantities than green legumes and chickpeas (Table 4). The caloric-adjusted median cooked consumption levels of the less consumed legume groups were 126.0 g/day for pigeon peas, 116.9 g*/*day for dry peas, 95.5 g/day for fava beans, 50.5 g/day for cowpeas, and 28.0 g/day for sprouted legumes in NHANES. We did not observe any change in consumption between 2011 and 2014 for consumption of legume groups, except for lentils, the intake level of which increased over time. The monthly average intake in legumes in the 2017 BLP survey is lower than the 2-day average intake in NHANES, which reflected the low consumption frequency of most legume groups. In the 2017 BLP survey (Table 4), 4.9% of participants ate at least 1 cup/day of cooked mature legumes, 16.8% at least 0.5 cup*/*day of cooked mature legumes, and 38.5% at least 37.5 g/day of cooked mature legumes during the last month. In comparison, 2.2% (2011–2012) and 1.8% (2013–2014) of NHANES participants ate at least 1 cup/day of cooked mature legumes, 7.1% (2011–2012) and 5.3% (2013–2014) at least 0.5 cup/day of cooked mature legumes, and 14.0% (2011–2012) and 10.7% (2013–2014) at least 37.5 g/day of cooked mature legumes during the 2 days of survey, after adjusting for caloric intake.

To compare legume consumers by cooked consumption level, we switched from “any legume” to “mature legume” consumption level, as dietary recommendations only exist for the latter. To identify characteristics that differentiate among mature-legume consumers, we compared marginal consumers (<37.5 g cooked mature legumes*/*day), recommended consumers (37.5–87.49 g/day), and beneficial amounts consumers (≥87.5 g*/*day). In NHANES (Table 5), young adults (18–35 years) ate less mature legumes, whereas older adults ate more mature legumes. Non-Hispanic white people ate lower amounts of mature legumes, whereas Mexicans and non-Hispanic Asians ate more mature legumes with intermediate amounts more prevalent for other Hispanics. Participants with >high school education or a higher income ate less mature legumes, whereas participants with <high school education and low income ate higher amounts. The caloric intake (Table 5) decreased with increased mature legume intake to levels below that of non-legume consumers (Table 1).

Higher mature legume intake was linked to a greater legume variety (Table 6). All mature legume consumers utilized dry beans and green legumes, although to different extents (higher in the recommended and disease prevention mature legume consumers versus the marginal low intake group). Recommended mature-legume consumers added chickpeas to their diet, and beneficial amounts legume consumers added lentils to their diet. Regardless of mature legume consumption level, mature legume consumers preferred the same dish types for the same legume groups (Table 6), which were dry beans for main dish or soup, green legumes or fava beans for side dish, chickpeas for dips, sprouted legumes for salad, and lentils, dry peas, or cowpeas for soup.

## 4. Discussion

The primary finding of both of our surveys (NHANES 2011–2014 and BLP 2017) is that daily legume consumption in US adults is rare, as less than 5% of participants in both surveys reported that they ate legumes each day. Approximately one-third of participants did not consume legumes within a month. In the companion study (BLP), we learned that primary reasons why people did not consume legumes were that people disliked their taste and texture, experienced digestive discomfort, or were concerned about their high carbohydrate content [20]. Consistent with the literature [5,12], non-consumers were more likely to be young adults or of non-Hispanic descent; however, neither age nor ethnicity appeared to play a role in their decision to not consume legumes [20]. In contrast, the lower price of legumes, especially during the winter months when other vegetables were more expensive, played a role in legume consumption and may explain in part why legume consumers were more likely to be of lower socio-economic status, as also observed in the literature [12]. Furthermore, legumes were a less expensive and a more environmentally sustainable protein source than meat and an alternative protein source for vegetarians, all of which were provided as reasons for consuming legumes in the BLP study [20].

We stratified mature legume consumers based on current US dietary recommendations for non-vegetarians, which are 1.5 cups/week of cooked mature legumes (equivalent to 37.5 g/day), which is equivalent to the recommendation of the healthy Mediterranean-style eating pattern [6]. With the same consumption level, Mitchell et al. [5] showed that 1.5 cups/week of mature legumes were insufficient to alter the nutrient profile, specifically the fiber and cholesterol content of the diet, and thereby did not provide health benefits. Instead, they proposed a cut-off of 0.5 cup/day of mature legumes (equivalent to 87.5 g/day), which we used as second cut-off. Approximately one-third of mature-legume consumers consumed mature legumes below the current dietary recommendations (marginal consumers). In other US studies, the median mature legume or dry bean consumption level usually falls into this category [17,21,22], which is consistent with our findings. Historically, dry legumes were not a staple in the US diet [2]. Mature legume consumption in the US was low in the 1960s, with 10 g per person, and bottomed out in the 1984, with 6 g per person [2]. Mature legume consumption increased by 1991 to 11 g per person, primarily due to an increase in consumption of other legumes than dry beans and has been stable since then [2]. Globally, the US is 100th out of 174 countries in legume consumption. In 2011, the leading legume consumers were Niger with 96 g per person and day, Rwanda with 82, United Arab Emirates with 66, and Cameroon, Nicaragua, and Tanzania with 56, 55, and 54, respectively [2].

Legume consumption patterns of marginal mature-legume consumers were weekly to monthly and lacked legume group variety, as they consisted of dry beans as main or side dish, green beans or peas as side dish or both. The primary dry bean types consumed in NHANES were pinto beans, black beans, kidney beans, and navy beans, which is similar to previously reported studies [5,12,17]; however, our findings suggest that black bean consumption has increased over the last decades. When asked for reasons for their low legume consumption, consumers cited problems with finding and preparing tasty recipes that include legumes; however, they did not question the health benefits of legumes, whereas non-legume consumers did [20]. In the Polyp Prevention Trial, participants remarked that it was difficult to choose legume-containing dish options, when eating in social situations [23]. This may explain in part why younger adults, non-Hispanic white people, and people at higher income to poverty ratio were over-represented in the marginal consumer group. Concerning is that dietary energy intake levels were higher in marginal mature-legume consumers than in non-legume consumers, which suggests that low legume consumption may do harm rather than benefit consumers. Marginal mature-legume intake levels were in line with the common nutritional concept of mature legumes, as part of the vegetable requirements. For example, the federal school meal regulations for receiving reimbursements requires 0.5 cups/week of mature legumes as part of the vegetable requirements [24]. This makes sense, as US dietary recommendations for mature legumes have been alternating over the last decades, between providing specific recommendations for legume consumption and including legumes in the vegetable recommendations [6,23], which can cause confusion in consumers. Similarly, the healthy Mediterranean-style eating pattern groups legumes as part of the vegetable group [6].

Another one-third of mature legume consumers ate mature legumes above current dietary recommendations, but below intake levels required to observe nutritional and health benefits [5]. Therefore, we defined them as recommended mature-legume consumers. Consistent with current US dietary recommendations and various Mediterranean-style plans, legume consumption patterns of recommended mature-legume consumers were typically weekly and included dry beans, green beans or peas, and chickpeas. A subset of recommended mature-legume consumers added lentils and sprouted legumes on a weekly basis to their legume mix. Recommended mature-legume consumers were a transitional group, as they in some aspects resembled more marginal mature-legume consumers, whereas in other aspects, they resembled more beneficial amounts mature-legume consumers. For example, when asking for reasons for their legume consumption pattern, they cited similar challenges as low legume consumers with finding and preparing healthy dishes with legumes [20]. In terms of age, demographics, income to poverty ratio and education levels, recommended mature-legume consumers were more similar to beneficial amounts of mature-legume consumers, whereas regarding ethnicity they were more similar to marginal mature-legume consumers. In regards to caloric intake, recommended mature-legume consumers had intermediate caloric intake; higher than marginal mature-legume consumers but lower than beneficial amounts mature-legume consumers, supporting the notion that recommended mature-legume consumption may be insufficient to provide the full nutritional and, thereby, health benefit.

Approximately a quarter of mature-legume consumers, or 15% of the total population, ate mature legumes at levels required to achieve nutritional and, thereby, disease prevention benefits, as supported by multiple population studies and meta-analyses, including ours [4,7,8,9,10]. We defined them as beneficial amounts mature-legume consumers. Legume consumption patterns of beneficial amounts legume consumers were typically daily or 3–4 times a week, which is consistent with US dietary recommendations for vegetarians, Dietary Approach to Stop Hypertension (DASH) plans, and Mediterranean-style plans that promote heavily legumes as partial replacement of animal-originating protein foods [6,25]. Regularly consumed legumes included dry beans, green beans or peas, chickpeas and lentils. A subset of this consumption group added sprouted legumes and dry peas on a weekly basis to their legume mix. Adding more lentils to the diet was the primary change from recommended to beneficial mature-legume consumption levels.

Beneficial amounts of mature-legume consumers cited digestive health benefits (i.e., high fiber, low cholesterol, and high vitamin content) and their taste and texture as primary reasons for their consumption [20]. Surprisingly, most of them were unaware that regular legume consumption may prevent obesity and chronic diseases [20]. Most beneficial amounts mature-legume consumers had mastered preparation of a variety of legume groups, which can explain partly why older adults and Mexicans were over-represented in the disease prevention group. It may also explain why in previous studies, mature legume consumption was higher in the US West and in rural areas [12]. Another important reason for high legume consumption was cost, as legumes were cheaper than other protein and vegetable sources, especially during times when fresh vegetables were expensive [20]. The lower legume cost could explain in part why adults of lower socioeconomic status were over-represented in the beneficial amounts group. Despite the fact that disease prevention consumers ate sufficient amounts of legumes, half of them expressed interest in increasing their legume consumption by diversifying their legume group and dish type choices (i.e., obtaining tasty recipes of less popular legume groups and dish types) [20]. Several BLP respondents responded that they lost body weight when they consumed legumes daily [20], which is consistent with the lower dietary energy intake of disease prevention mature-legume consumers compared with the other groups in 2011–2014 NHANES and findings in the Legume Inflammation Feeding Experiment (LIFE) study, in which participants lost on average 1 kg body weight per week on a high dry bean (navy, pinto, kidney and black bean) diet over four weeks [26].

The strengths of this study included a large sample size with a nationally representative sample. The NHANES has carefully controlled protocols and screens 24-h dietary recalls to confirm they are valid and complete; as stated, the NHANES also uses the multiple pass method to obtain dietary intake [14]. However, NHANES is not without limitations. It is a cross-sectional study, and thus, causality cannot be established. Additionally, two 24-h dietary recalls do not represent one’s habitual dietary intakes; therefore, we queried in the 2017 BLP survey the legume consumption pattern. Additionally, 24-h dietary recalls have several intrinsic limitations. They are memory dependent and over- or under-reporting of intake may occur [14]. In NHANES, changes in legume related food codes occurred over different year cycles, which made itdifficult to compare yearly consumption over time and, thereby, this can affect the quantification of legume consumption. There is a variety of ways how legumes are grouped in the literature and defined by governments [1,5,6,12,15,17,18,19,27], which also makes comparisons difficult and causes confusion for consumers and scientists alike. We chose to combine mature and immature legumes (excluding high lipid legumes) that were similar in nutrient and disease prevention profile [17] and disregard USDA guidelines that group members of the botanical legume family into six categories [6].

There are several limitations to the BLP online survey. The participants were parents or guardians of families in the Corvallis, Oregon, area that were interested in nutrition and health; thus, our ability to generalize the finding from the study could be limited and our findings may be region-specific; however, the observed legume consumption patterns in BLP and NHANES are similar, suggesting the generalizability of our BLP findings. Data collection occurred over a 4-week period in November and December, which may have impacted results; however, based on our survey results, seasonal variability in legume availability was not a consumer concern. To keep the duration of the survey below 10 min, we did not ask for the average caloric intake of participants. Adjusting for caloric intake had little impact on legume consumption quantities and proportion of legume consumption groups in NHANES, and, therefore, is unlikely to have affected results in the BLP survey. Another potential concern is that the BLP survey was not validated; however, categories for legume consumption frequency and amounts and legume group and dish type were adapted from validated NIH surveys [16,17] and the FAO guidelines [18], respectively. To reach a large target population, we chose direct e-mail messages, despite their limitations as unused and incorrect email addresses might have diminished the accuracy of the listserv and survey participation [28]. We did not use any incentives for participation, which might have resulted in a higher response rate. To preserve the anonymity of the respondents, we did not collect any demographic characteristics related to respondents. Furthermore, in order to keep the BLP survey short and increase the response rate, we did not query in either survey for physical activity level or other food choices of the consumer groups. Hence, we were not able to explore these aspects, which may explain in part some of our results. Thus, subgroups of consumers may differ in their benefits, barriers and preferences for legume consumption, which needs to be explored in future studies.

## 5. Conclusions

In conclusion, although peer-reviewed research demonstrated that regular legume consumption can prevent obesity, metabolic syndrome, cardiovascular diseases and colorectal cancer, regular legume consumption is low in US adults and the current level of consumption is unlikely to confer any nutritional and health benefits. Limited knowledge about strategies to successfully incorporate legumes into the diet may refrain consumers from eating sufficient amounts of legumes to receive nutritional and health benefits. Furthermore, one-third of the population did not consume legumes during the last month. There is a disconnect between known nutritional and health benefits of legumes, current dietary recommendations for legumes, and current legume consumption patterns. This disconnect raises questions on the effectiveness of the current efforts in providing consumers information about the nutritional and health benefits of legumes and how to incorporate a variety of legume types into their diet. We recommend that future legume information materials focus on a) highlighting the specific disease prevention benefits of legumes in addition to their nutrient content and suggestions on how to prevent gastro-intestinal discomfort and b) featuring the variety of legume types and dishes with their unique flavor and textural characteristics and preparation techniques. Displaying legume as a food group and highlighting the variety of legumes on MyPlate is an important first step and will help consumers understand why legumes are an important food group, with dietary recommendations.

## Figures and Tables

**Table 1:** Comparison between legume consumers and non-consumers: National Health and Nutrition Examination Survey (NHANES) and Beans, Lentils, Peas Nutrition Education Online Parent Survey (BLP) ^1^.

Characteristics	NHANES (2011–2014)			BLP (2017) ^2^		
	Any Legume Consumers (within 2 Days)			Any Legume Consumers (within Last Month)		
	Yes	No	*p*-Value	Yes	No	*p*-Value
	***n* = 3215**	***n* = 8640**		***n* = 89**	***n* = 52**	
**Age (year)**						
18–25	10.4	16.5	<0.0001	1.1	7.5	0.26
26–35	15.7	16.4		15.7	18.9	
36–45	17.0	18.2		56.2	43.4	
46–55	20.3	17.3		21.3	26.4	
56–65	18.0	16.5		3.4	3.8	
66–75	11.7	8.6		2.2	0	
76+	6.9	6.6		0	0	
**Gender**						
Female	51.7	51.0	0.62	84.6	86.5	0.76
Male	48.2	49.0		15.4	13.5	
**Ethnicity (NH = Non-Hispanic)**						
NH Asian	5.2	5.6	<0.0001			
NH Black	10.8	11.8				
NH White	61.5	68.0				
Mexican	11.6	7.6				
Other Hispanic	8.0	5.4				
Other	3.0	2.6				
**Education**						
<High school	18.2	16.2	<0.0001			
High school	18.0	22.7				
>High school	63.7	61.1				
**Income to poverty ratio**						
<1	16.4	18.0	0.18			
>1	83.6	82.0				
**Dietary Energy (kcal/day)**	2092 (15)	2051 (10)	0.02			

^1^ Values are percent (%) of total population except for dietary energy, which is average kcal per day with standard errors in parenthesis. Any legumes include mature, green, and sprouted legumes. In NHANES, any legume consumers refers to legume consumption on day 1 or 2 of the survey, similar to 3–4 days per week consumption necessary for nutritional and health benefits. In BLP, any legume consumers refers to any legume consumption within the last month. ^2^ Ethnicity, education, income and total caloric intake were not collected in BLP.

**Table 2:** Consumption frequency of legume types in US adults: National Health and Nutrition Examination Survey (NHANES) and Beans, Lentils, Peas Nutrition Education Online Parent Survey (BLP) ^1^.

Survey (Year)	Any Legume ^2^	Mature Legume	Dry Beans	Green Legume	Chickpeas	Lentils
**NHANES (2011–2012; *n* = 5807)**						
Day 1	18.5	12.8	10.0	6.3	1.6	1.1
Day 2	16.5	10.7	9.6	6.4	0.9	0.9
Each day ^3^	5.6	3.5	2.4	0.9	0.09	0.3
Over 2 days ^4^	29.3	20.0	16.9	11.8	2.3	1.7
**NHANES (2013–2014; *n* = 6048)**						
Day 1	15.4	9.7	7.4	6.1	1.1	1.2
Day 2	13.7	8.3	6.5	5.5	1.0	0.6
Each day	4.0	2.5	1.6	0.9	0.2	0.3
Over 2 days	25.0	15.4	12.4	10.9	1.9	1.2
**BLP (2017; *n* = 143)**						
Each day	4.9	4.2	4.2	1.4	0	0
Over 2 days	23.1	18.9	18.2	7.7	5.6	2.1
Weekly	57.3	50.3	46.9	39.2	29.4	12.6
Last month	63.6	62.2	56.6	53.8	51.0	41.3

^1^ Values are percent (%) of total population. ^2^ Total refers to green beans/lentils/peas, sprouted beans/lentils/peas, dry beans, fava beans, dry peas, chickpeas, pigeon peas (only in NHANES), and lentils. Mature legumes exclude the green and sprouted legume types. Legume types not shown are cowpeas, dry peas, fava beans, and pigeon peas, each of which was reported by less than 0.4% of NHANES participants. ^3^ Each day means consumption on day 1 and 2 in NHANES and daily consumption in BLP. ^4^ Over 2 days means consumption on day 1 or 2 in NHANES and at least 3–4 days per week consumption in BLP.

**Table 3:** Consumption frequency of legume-containing dish types in US adults: National Health and Nutrition Examination Survey (NHANES) and Beans, Lentils, Peas Nutrition Education Online Parent Survey (BLP) ^1^.

Survey (Year)	Side Dish ^2^	Main Dish	Soups	Dips	Salads
**NHANES (2011–2012; *n* = 5807)**					
Day 1	12.8	3.9	1.4	1.2	0.2
Day 2	12.5	3.0	0.9	0.6	0.2
Each day ^3^	2.0	0.7	0.2	0.05	0
Over 2 days ^4^	24.9	6.3	2.2	1.7	0.4
**NHANES (2013–2014; *n* = 6048)**					
Day 1	12.6	1.3	0.6	1.0	0.3
Day 2	11.4	0.9	0.4	0.9	0.2
Each day	2.9	0.1	0.09	0.2	0
Over 2 days	20.8	2.1	1.0	1.7	0.6
**BLP (2017; *n* = 143)**					
Each day	2.1	2.8	0.7	0	0.7
Over 2 days	6.3	14.7	4.2	2.8	1.4
Last month	47.6	80.4	67.8	34.3	25.9

^1^ Values are percentage (%) of total population. ^2^ Side Dish in NHANES referred to codes that had legumes as sole descriptor or combined with another vegetable. Main Dish in NHANES referred to codes that include legumes as part of a main dish (combined with rice/meat/stew/chili). Soups referred that include legumes as part of a soup. Dips referred to codes that include hummus or legumes as part of a dip or sauce. Salads referred to codes that include legumes as part of a salad. Dish types not shown are breakfast items, snacks, baked goods, and dessert, each of which was reported by less than 0.2% of NHANES participants. ^3^ Each day means consumption on day 1 and 2 in NHANES and daily consumption in BLP. ^4^ Over 2 days means consumption on day 1 or 2 in NHANES and at least 3–4 days/week consumption in BLP.

**Table 4:** Legume consumption levels (in g cooked legumes/day) in legume-consuming US adults: National Health and Nutrition Examination Survey (NHANES) and Beans, Lentils, Peas Nutrition Education Online Parent Survey (BLP) ^1^.

Survey (Year)	Any Legume ^2^	Mature Legume	Dry Beans	Green Legumes	Chickpeas	Lentils
**NHANES (2011–2012; *n* = 5807; 2-day average)**						
25% Percentile	32.4	31.7	31.6	25.5	15.4	54.0
Median	59.3	63.3	59.8	40.0	31.7	93.0
75% Percentile	98.0	111.1	99.5	67.5	61.5	156.6
**NHANES (2013–2014; *n* = 6048; 2-day average)**						
25% Percentile	31.8	32.7	33.2	28.5	21.1	80.3
Median	55.5	63.3	59.1	41.2	30.8	129.2
75% Percentile	91.4	111.0	94.9	67.5	61.5	186.7
**BLP (2017; *n* = 143; during the last month)**						
25% Percentile	40.9	23.2	12.5	6.3	3.9	3.1
Median	74.9	56.8	26.4	12.5	12.5	7.2
75% Percentile	117.3	87.7	52.9	43.8	14.5	16.5
**Adjusted to 2000 kcal**						
**NHANES (2011–2012; *n* = 5807; 2-day average)**						
25% Percentile	31.0	32.3	31.3	25.3	14.3	63.5
Median	59.3	61.6	57.5	41.3	31.7	98.2
75% Percentile	105.9	112.0	101.6	74.4	64.0	163.2
**NHANES (2013–2014; *n*= 6048; 2-day average)**						
25% Percentile	31.3	31.9	32.0	26.5	16.8	81.9
Median	57.2	59.8	57.8	47.3	33.0	139.8
75% Percentile	101.1	110.0	98.0	76.1	56.8	210.1

^1^ Values are median consumption in g cooked legumes/day for those who consumed the specific legume type. Participants that did not consume the specific legume type were not included in the values ^2^ Total refers to green beans/lentils/peas, sprouted beans/lentils/peas, dry beans, fava beans, dry peas, chickpeas, pigeon peas (only in NHANES), and lentils. Mature legumes exclude the green and sprouted legume types. Legume types not shown are cowpeas, dry peas, fava beans, and pigeon peas, each of which was reported by less than 0.4% of NHANES participants.

**Table 5:** Demographic characteristics among mature-legume consumers, stratified by dietary recommendations for cooked mature legume consumption (37.5 g/day) and amounts demonstrating nutritional and disease prevention benefits (87.5 g/day): National Health and Nutrition Examination Survey (NHANES) ^1^.

Characteristics	Cooked Mature Legume Consumption Level			
	Marginal	Recommended	Beneficial	*p*-Value
	0.1–37.5 g/day	37.5–87.5 g/day	≥ 87.5 g/day	
	***n* = 637**	***n* = 601**	***n* = 861**	
**Age (year)**				
18–25	13.0	12.8	8.9	0.0005
26–35	19.8	14.3	13.6	
36–45	18.3	16.3	18.2	
46–55	19.0	20.3	18.1	
56–65	16.1	18.1	18.9	
66–75	9.7	12.2	14.6	
76+	4.1	6.0	7.6	
**Gender**				
Female	52.4	49.3	49.3	0.41
Male	47.6	50.8	50.8	
**Ethnicity (NH = Non-Hispanic)**				
NH Asian	9.9	9.6	16.4	<0.0001
NH Black	17.3	16.1	16.8	
NH White	33.8	31.0	24.3	
Mexican	21.5	20.9	24.6	
Other Hispanic	15.7	19.4	15.1	
Other	1.9	3.2	2.8	
**Education**				
<High school	25.4	26.4	33.3	0.008
High school	19.7	18.3	16.7	
>High school	54.9	55.3	50.1	
**Income to poverty ratio**				
<1	21.5	26.3	26.5	0.07
>1	78.6	73.8	73.5	
**Dietary Energy (kcal/day)**	2371 (33)	2129 (34)	1973 (29)	<0.0001

^1^ Values are percent (%) of mature-legume consumer group, except for dietary energy, which is average kcal/day with standard errors in parenthesis. Mature legume consumption was adjusted by caloric intake.

**Table 6:** Legume consumption patterns among mature-legume consumers stratified by dietary recommendations for cooked mature legume consumption (37.5 g/day) and amounts demonstrating nutritional and disease prevention benefits (87.5 g/day): Beans, Lentils, Peas Nutrition Education Online Parent Survey (BLP).

Characteristic	Cooked Mature Legume Consumption Level			
	Marginal	Recommended	Beneficial	*p*-Value
	0.1–37.5 g/day	37.5–87.5 g/day	≥87.5 g/day	
	***n* = 32**	***n* = 32**	***n* = 23**	
**Important dietary legume source ^1^**				
Dry Beans	50.0	93.8	95.7	<0.0001
Green Legumes	37.5	62.5	60.9	0.10
Chickpeas	9.4	59.4	69.6	<0.0001
Lentils	3.1	25.0	65.2	<0.0001
Sprouted Legumes	3.1	21.9	21.7	0.06
Dry Peas	0	3.1	17.4	0.02
Cowpeas	0	9.4	0	0.10
Horse Beans	0	3.1	0	1
**Preferred dish type for legume source ^2^**				
Dry Beans—Main/Soup	90.0	87.1	90.9	1
Green Legumes—Side	64.3	67.9	52.4	0.58
Chickpeas—Dip	60.0	58.1	65.0	0.91
Lentils—Soup	70.6	52.2	60.0	0.54
Sprouted Legumes—Salad	42.9	61.5	60.0	0.80
Dry Peas—Soup	60.0	100	60.0	0.02
Cowpeas—Soup	25.0	50.0	33.3	0.72
Horse Beans—Side	50.0	50.0	0	1

^1^ Values are percentage (%) of mature-legume consumer group who consumed legume source at >87.5 g/wk.^2^ Values are percent (%) of mature-legume consumer group who indicated dish type for legume source as primary dish type.
